# High-grade endometrial stromal sarcoma heterogeneity: Fusion-driven, fusion-negative, and related undifferentiated uterine sarcomas

**DOI:** 10.1073/pnas.2609538123

**Published:** 2026-06-18

**Authors:** Jan Hojný, Michaela Kendall Bártů, Radoslav Matěj, Pavel Dundr

**Affiliations:** ^a^ Department of Pathology, First Faculty of Medicine, Charles University and General University Hospital in Prague, Prague 128 00, Czech Republic; ^b^ Department of Pathology, 3rd Faculty of Medicine, Charles University in Prague and University Hospital Kralovske Vinohrady, Prague 100 34, Czech Republic; ^c^ Department of Pathology and Molecular Medicine, 3rd Faculty of Medicine, Charles University in Prague and Thomayer University Hospital, Prague 140 59, Czech Republic

**Fig. 1.**
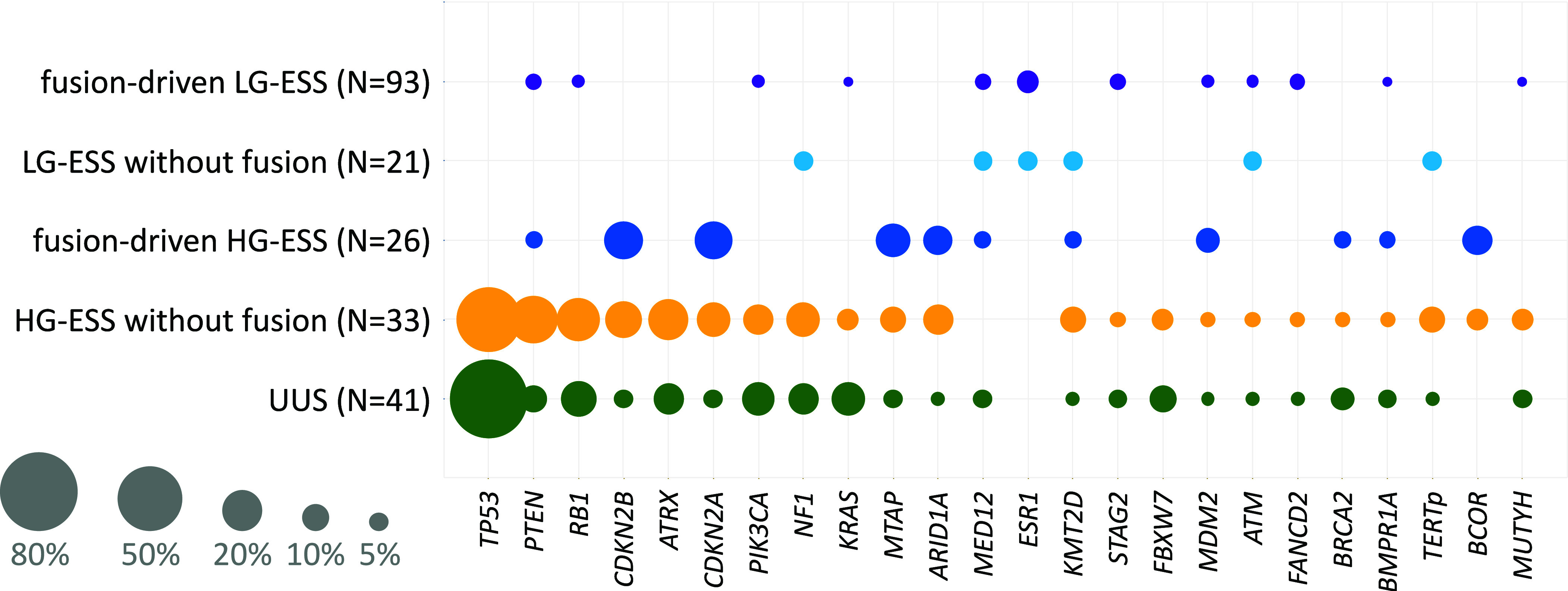
Bubble plot illustrating the spectrum and frequency of genes with DNA-level aberrations (mutations and copy number variants) in endometrial stromal sarcomas, based on the supplementary data from Dundr et al. (6). Bubble diameter indicates the frequency of altered cases in each cohort. LG-ESS, low-grade endometrial stromal sarcoma; HG-ESS, high-grade endometrial stromal sarcoma; UUS, undifferentiated uterine sarcoma. Only genes altered in at least five samples are shown.

We read with great interest the study by Hartwich et al. presenting an integrated WGS/WES/RNA-seq analysis of 80 endometrial stromal sarcomas (ESS) and reporting *RAD54B* amplification as a promising oncogenic driver associated with worse survival (1). They further described complex chromosomal alterations in high-grade ESS (HG-ESS) and RNA-seq-based fusion landscape in the subset evaluable for fusion calling. Their results are impressive and bring important insights into potentially targetable alterations, including MSI-altered and *POLE*-mutated cases. Based on current knowledge, HG-ESS and undifferentiated uterine sarcomas (UUS) are clinically aggressive uterine mesenchymal neoplasms with limited treatment options. These tumors are diagnostically challenging because similar high-grade morphology can be driven by distinct molecular alterations. Over the last decade, recurrent gene fusions defined multiple HG-ESS types, including *YWHAE* rearrangements, *BCOR*-alterations (e.g., *ZC3H7B::BCOR*, *BCOR*-internal tandem duplications), *BCORL1* rearrangements, and the more recently proposed *KDM2B* rearrangements (2–5). These fusion-driven tumors show a characteristic molecular profile with relatively low secondary mutation burden (6). On the other hand, HG-ESS lacking recurrent fusions (“fusion-negative”) exhibit more often a mutation-driven landscape enriched for alterations in *TP53*, *RB1*, *PTEN*, *ATRX*, *PI3K* pathway genes, partially overlapping with UUS, and, moreover, may show different clinical behavior compared with fusion-driven HG-ESS (Fig. 1) (6). These biological differences in HG-ESS are further supported by a recently published unsupervised RNA-Seq clustering of 262 ESS cases in which fusion-driven HG-ESS form a distinct expression group, while fusion-negative monomorphic HG-ESS show substantial transcriptional overlap with pleomorphic UUS and further separates into mixed clusters differing mainly by immune activity, with survival implications (7).

Hartwich et al. in their study delineated several genomic distinctions between low- and high-grade ESS, which are highly informative. However, it would be of interest to know whether the key mutational and signature-based inferences in HG-ESS were stratified by fusion status in their study. Emerging data suggest that fusion-negative HG-ESS and UUS have similar prognostic significance and thus differ from fusion-driven HG-ESS. Such stratification could contribute significantly to the interpretation of their conclusions. Additionally, the authors identified a hypermutated subset of HG-ESS/UUS with *POLE* or mismatch-repair alterations suggestive of potential immunotherapy responsiveness. These findings would be of significant clinical impact as hypermutated and/or ultramutated cases can indeed be responsive to immunotherapy. However, such findings in this cohort are rather surprising considering previous studies. In our previous cohort on 379 uterine sarcomas, undifferentiated carcinoma (typically MSI-altered or *POLE*-mutated) was a common mimic of HG-ESS/UUS (7.6%; 29/379 cases); a similar proportion to the MSI/*POLE*-mutated cases reported in the Hartwich study (6). As such it would be of great interest to know the details of the morphology and immunohistochemical findings of MSI/*POLE*-mutated cases described in this cohort.

Finally, we congratulate the authors on assembling a rare and clinically important ESS cohort. We believe that fusion-stratified analyses in high-grade disease, and more detailed characterization of the *POLE*/MMR-altered hypermutated cases, would strengthen interpretability and facilitate the translation of these findings into routine diagnostics.
